# Microfluidic co-cultures of retinal pigment epithelial cells and vascular endothelial cells to investigate choroidal angiogenesis

**DOI:** 10.1038/s41598-017-03788-5

**Published:** 2017-06-14

**Authors:** Li-Jiun Chen, Shuntaro Ito, Hiroyuki Kai, Kuniaki Nagamine, Nobuhiro Nagai, Matsuhiko Nishizawa, Toshiaki Abe, Hirokazu Kaji

**Affiliations:** 1 Department of Finemechanics, Graduate School of Engineering, Tohoku University, Sendai, Japan; 2 Division of Clinical Cell Therapy, United Centers for Advanced Research and Translational Medicine, Tohoku University Graduate School of Medicine, Sendai, Japan

## Abstract

Angiogenesis plays a critical role in many diseases, including macular degeneration. At present, the pathological mechanisms remain unclear while appropriate models dissecting regulation of angiogenic processes are lacking. We propose an *in vitro* angiogenesis process and test it by examining the co-culture of human retinal pigmental epithelial cells (ARPE-19) and human umbilical vein endothelial cells (HUVEC) inside a microfluidic device. From characterisation of the APRE-19 monoculture, the tight junction protein (ZO-1) was found on the cells cultured in the microfluidic device but changes in the medium conditions did not affect the integrity of monolayers found in the permeability tests. Vascular endothelial growth factor (VEGF) secretion was elevated under low glucose and hypoxia conditions compared to the control. After confirming the angiogenic ability of HUVEC, the cell-cell interactions were analyzed under lowered glucose medium and chemical hypoxia by exposing ARPE-19 cells to cobalt (II) chloride (CoCl_2_). Heterotypic interactions between ARPE-19 and HUVEC were observed, but proliferation of HUVEC was hindered once the monolayer of ARPE-19 started breaking down. The above characterisations showed that alterations in glucose concentration and/or oxygen level as induced by chemical hypoxia causes elevations in VEGF produced in ARPE-19 which in turn affected directional growth of HUVEC.

## Introduction

Angiogenesis, the growth of new capillary blood vessels from pre-existing vascular structures, occurs naturally in the body during reproduction and wound healing. The process is regulated by a fine balance between growth and inhibitory factors in healthy tissues. However, if the balance is disturbed, abnormal blood vessel growth could lead to debilitating conditions including cancer, cardiovascular disease, stroke and many more. Pathological angiogenesis of the retina is one of the key factors of irreversible causes of blindness as observed in diabetic retinopathy, age-related macular degeneration and retinopathy of prematurity^1, 2^. In the case of the more advanced type of age-related macular degeneration (wet AMD), abnormal blood vessels develop under the macula and compromise Bruch’s membrane, leading to leakage of fluid (exudate) or blood. According to the Age-Related Eye Disease Study (AREDS), 1.7% of population over 55 years old in the United States are affected by AMD, and 12% of the patients have developed neovascular AMD^3^. Not limited to the United Sates, AMD is the leading cause of legal blindness in individuals over 65 years old in the developed world^4^. Choroidal neovascularization of wet AMD occurs in response to the abnormal secretion of growth factors, of which vascular endothelial growth factor (VEGF) being the most important mediators of angiogenesis. VEGF-A belongs to a gene family that includes VEGF-B, VEGF-C, VEGF-D, VEGF-E and placental growth factor (PlGF); it is a secreted growth factor peptide that promotes vascular endothelial cell proliferation, migration and tube formations^5^. Studies have demonstrated the efficacy and safety of the anti-VEGF agents bevacizumab (Avastin; Genentech/Roche), ranibizumab (Lucentis; Genetech/Roche) and pegaptanib (Macugen; EyeTech, Inc) in the treatment of retinal disorders^5^. The biologics are delivered via an intravitreal injection where the medicine is injected into the vitreous near the retina at the back of the eye. An intravitreal injection is an intraocular operation; infections and devastating complications arise if the procedure is not administered properly^6^. Regarding anti-VEGF treatments, there are mixed views on their side-effects and complications^5, 7, 8^, and re-treatments are required. The inconvenience and cost that result from monthly injections increase the burden on patients as well as the health care system^4^. Regardless of the downsides of the anti-VEGF treatment, treatment only limits vision loss by inhibition of vascular leakage but does not address disease pathogenesis^4^. Therefore, the underlying mechanisms that cause the blood vessels to invade remain unclear; while there are studies focusing on alterations in the microenvironment of RPE cells, there are other studies investigating the molecular aspects that suggest the role of the DNA damage-repair system in the mitochondria as the cause of early pathological AMD^4, 9^. Choroidal neovascularization is promoted and exacerbated when there are changes in the extracellular microenvironment *in vivo*, especially hypoxia, inflammation or oncogene products which lead to the upregulation of growth factors, integrins and proteinases, resulting in the formation of new vessels^10, 11^.

Angiogenesis has been extensively studied by using co-culture models; for example, angiogenic conditions have been characterised in 2D culture insert plates^10, 12–14^. However, in many of the studies, behaviour of a single cell type (mostly endothelial cells) was observed through the use of conditioned media, i.e., different types of cells were not cultured at the same time. Since hydrogels have increased in popularity, various studies have demonstrated their possible applications on cell cultures and forming microchannels^15–19^, thus diversifying research direction.

Recently, much attention is on lab-on-a-chip technology which is widely applied in various fields of studies. The size of a chip typically ranges from a few to hundreds microns. Having the advantages of minimizing the resources and working in parallel, it is being used in studying surface patterning, flow shear stress and physiology of tissues, etc. Recent reviews have succinctly summarized a variety of assays in organs-on-chips, detailing possible applications with the technology^20, 21^. By culturing cells within controlled fluidic environments, microfluidic devices serve as an alternative to animal models with no ethical issues and are capable of identifying diagnostic markers and developing drug regimens, without cross-interactions between different pathways likely found in animal models.

In this study, we build a simplified microfluidic co-culture model of the ocular fundus tissue in an attempt to elucidate AMD pathology. The retinal cells, Bruch’s membrane and the choroid are represented by human retinal pigmental epithelial (ARPE-19) cells, a porous membrane and human umbilical vein endothelial cells (HUVEC). The platform enables long-term cell culture and imposing angiogenic conditions *in vitro* where we investigated changes of RPE microenvironments, the effects of glucose concentration and chemical hypoxia on cell-cell interactions. We believe we are one of the few groups who have developed an *in vitro* co-culture of the ocular fundus model in microfluidic devices to examine angiogenesis. Not only can cell-cell interactions be observed, the microfluidic system provides a more physiologically realistic environment compared to static culture insert plates. The microdevice can be fabricated easily in a short amount of time; with the same fabrication methods and slight alteration of the design, the microfluidic system can be tailored to other applications, thus demonstrating a great potential in medical diagnosis and pharmacokinetics.

## Results and Discussion

### Microfluidic co-culture platform design

**Figure 1:**
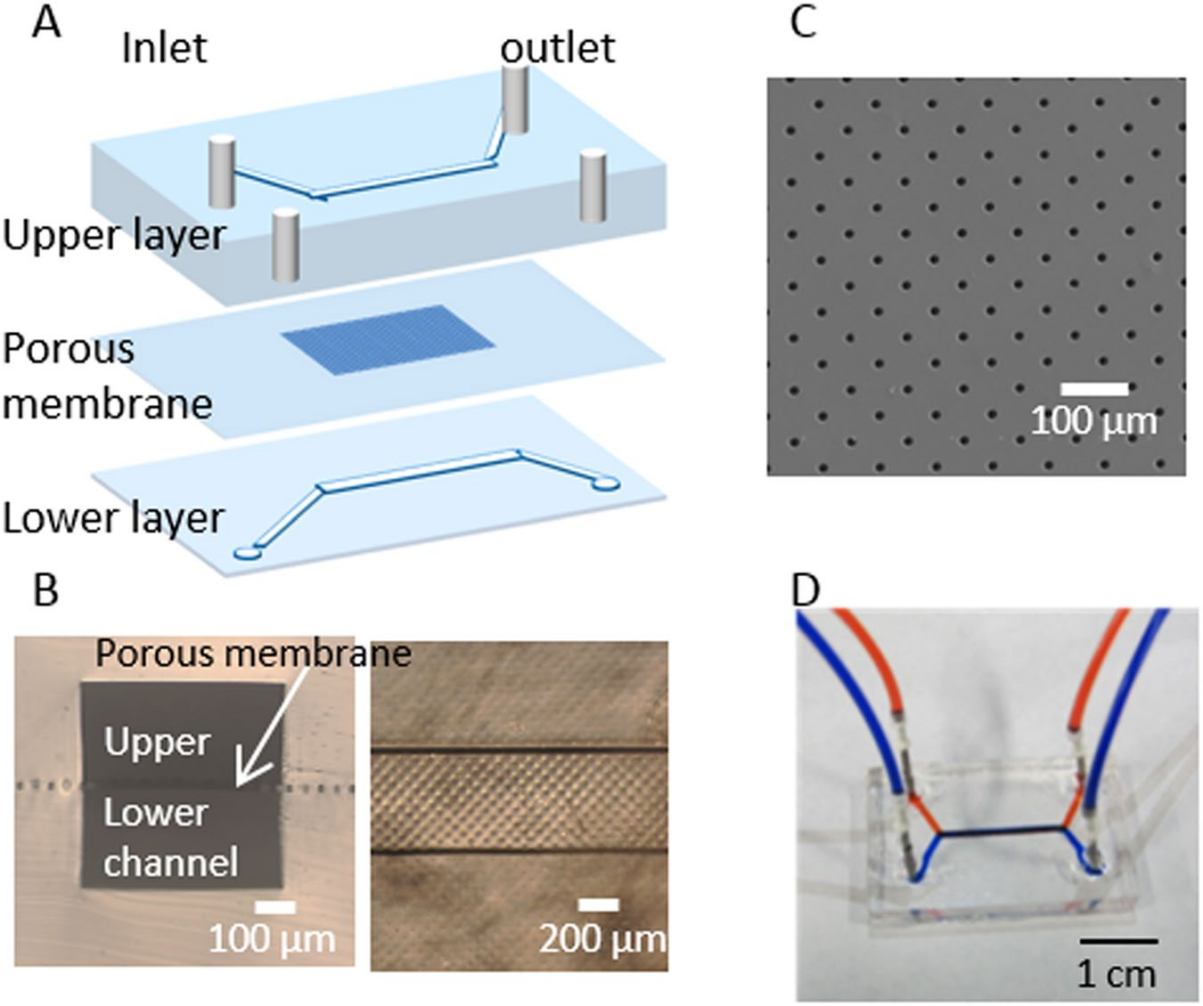
Microfluidic device configuration. (**A**) Exploded view and schematic. (**B**) Cross-section (left) and top (right) view of the device. (**C**) SEM image of the porous membrane. (**D**) Photograph of a device with dyes in fluidic channels.

We have examined responses of cells in a logical way, starting from characterising ARPE-19 and HUVEC individually before examining the co-culture under different conditions. The device is designed in such a way that ARPE-19 cells and HUVEC are separated by a porous membrane, similar to the *in vivo* anatomy where retinal cells are separated from the choroids by Bruch’s membrane (BM) (Fig. 1). Bruch’s membrane supports fundamental cellular functions and cell-cell communication while being formed and maintained from both the RPE cells and choroids in a coordinated fashion^22^. Cells in our device are separated by a fibronectin-coated 6.5 μm-thick membrane, which is also permeable, strong, flexible, biologically inert and able to support the functions of RPE cells, fulfilling the requirements of being an ideal prosthetic Bruch’s membrane^23^. However, the PDMS membrane cannot dynamically mimic the thickening process (drusen depositing) as in dry type AMD patients. It also lacks the 3D environment to support differentiation of cells and the capillary tube formation. As a result, we used endothelial cell migration as a surrogate for the initial stage in angiogenic tube formation.

As the pore diameter of the porous membrane (10 µm) is comparable to floating ARPE-19 and HUVEC cells, extra precaution was made to ensure that cells attached only on the desired side of the membrane.

### Characterisation of ARPE-19: VEGF collection and evaluation of barrier function

**Figure 2:**
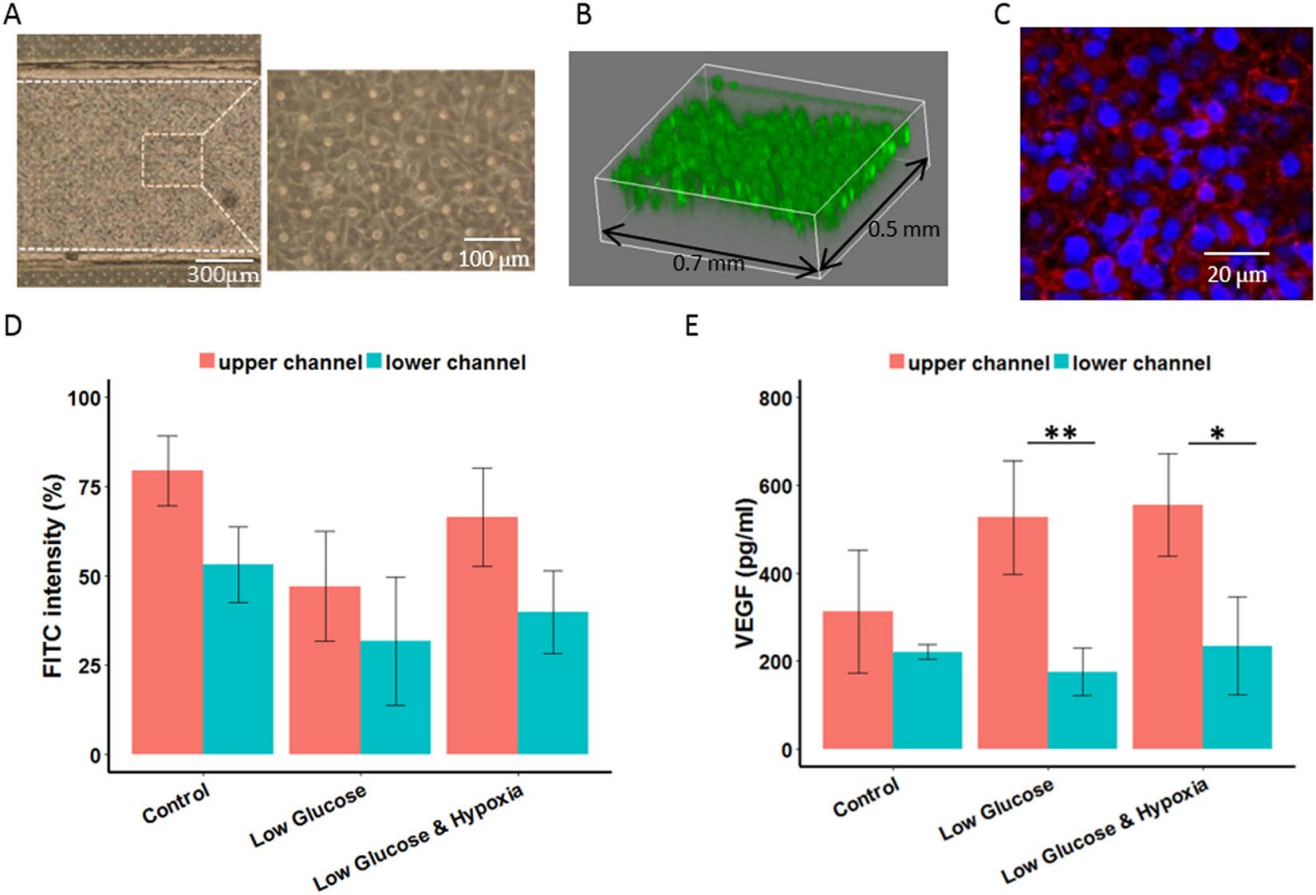
Characterisations of ARPE-19 cells. (**A**–**C**) Qualitative assessment of the monolayer inside a microfluidic device. (**A**) Optical microscopic image, (**B**) 3D confocal microscopic image (cell tracker green), and (**C**) fluorescent image (blue: nucleus; red: ZO-1). (**D**,**E**) Plots of permeability to FITC-Dextran and concentrations of VEGF secretion under different conditions imposed by altering glucose concentration and/or adding CoCl_2_ to the medium. Data sets were analyzed using 2-way ANOVA tests (showing a significant interaction effect for (**E**), p = 0.04); the asterisks * indicate significant findings from Tukey’s post-hoc tests. (**D**) mean FITC intensity (%) from lower channels is significantly different than those from upper channels (*p = 0.0005); pairwise comparisons between conditions are insignificant. (**E**) Pairwise comparisons between experimental conditions are insignificant while pairwise comparisons among the condition (red and blue bars for low glucose and low glucose & hypoxia) are significant (**p = 0.0022 and *p = 0.0162, respectively). N = 9 for (**D**) and (**E**).

In an attempt to characterise ARPE-19 in the microfluidic device, we first sought to confirm the presence of the intercellular tight junction (TJ) of RPE by examining the permeability of ARPE-19 monolayer to FITC dextran in addition to the visual assessment of the growth of cells inside the microfluidic device (Fig. 2A–C). TJs serve as an indication on how tight the barrier function is and is of particular importance in the blood-retina barrier (BRB). As the outer BRB prevents transport of molecules larger than 300 kDa into and out of the retina^22^, we do not anticipate 70 kDa FITC-dextran to be obstructed by monolayers in our permeability test.

Results show that the concentrations of FITC-dextran were significantly higher in the upper channel compared to the lower channel among the experimental conditions (p = 0.0005, see also Fig. 2D). There was an overlap in results between the upper and lower channels in the low glucose case, but the differences between different conditions were not statistically significant. This implied that the monolayers remained relatively stable under low glucose and/or hypoxic conditions. The results agreed with the TEER from conventional tissue culture treated culture insert plates in which no significant differences in resistance values between all conditions were found (Fig. S2).

In addition to identifying the presence of TJs, we measured the VEGF concentration secreted by the monolayer. Innate RPE cells secrete VEGF predominantly from the basolateral side to help regulation of the metabolism of the choroid vessels^24, 25^. It has been reported previously that RPE cells are prone to secrete an even higher concentration of VEGF if there is slight alteration to the microenvironment that induces DNA damage as non-lethal levels of DNA damage are postulated to activate stress-induced premature cellular senescence (SIPS) of cells^26^. Increasing non-hexagonal RPE cell shapes, reminiscent of the SIPS characteristic alterations of cell shape, has been described in aging retinas and AMD donor eyes^26^. We resorted to using CoCl_2_ to mimic hypoxia due to the limited numbers of incubators available and also considered the fact that the cobalt chloride method is not only an inexpensive way in inducing HIF-1 alpha production, but also avoids oxygen re-entering the chamber every time images were taken which might affect the results^27, 28^. We finalized the concentration of CoCl_2_ to be 150 µM, the non-lethal hypoxic concentration after determining viability of ARPE-19 cells under CoCl_2_ of 100–300 µM (data not shown).

From the VEGF outputs (Fig. 2E), ARPE-19 responded to lowered glucose and hypoxic microenvironments by increasing VEGF secretion (68.1% and 77.1%, respectively, compared to the control in the upper channel). Studies have found that the expression of VEGF-C and VEGFR-3 is upregulated in ARPE-19 cells after being exposed to hypoxia *in vitro*, consistent with the increased concentration of VEGF detected in the extracellular medium^10^. We also analysed VEGF from cells cultured in transwell culture plate inserts (Fig. S3). Both sets of results did not show basolateral-dominant secretion, but align with a study that found net secretion from the apical side of the cells^29^. Another study pointed out that polarized secretion was toward the apical side only when RPE cells were exposed to light damage^24^. There are many possible factors contributing to these observations including density of the porous membrane, origin of the cells and whether it is a cell line. Although ARPE-19 cell line is widely used as a model of human RPE cell function, there are discrepancies in current standard practices in culturing ARPE-19 cells, i.e. the use of defined media formulations, porous supports permitting separation of apical and basal media compartments, and extended culture periods for maturation^23, 30^. *In vivo* retinal pigment epithelium (RPE) expresses RPE-specific markers CRALBP and RPE65. In addition, it has defined cell borders, an overall cobblestone appearance and noticeable pigmentation^31^. As the ARPE-19 cells used in our experiments did not display features characteristic of RPE despite the long-term culturing, we suspected that the cells were not fully differentiated which added on as another factor contributing to the unexpected behaviour.

### Characterisation of HUVEC: migration

In order to characterise the behaviour of HUVEC inside the microfluidic device, we experimentally supplied VEGF to the upper channel of the device. This approach is comparable to the well-known Boyden assay except cells were under a constant flow inside the device. HUVEC were seeded at the lower channel of the device; we interpreted that directional migration of cells occurred when HUVEC moved across the porous membrane to the upper channel under the presence of VEGF gradient.

**Figure 3:**
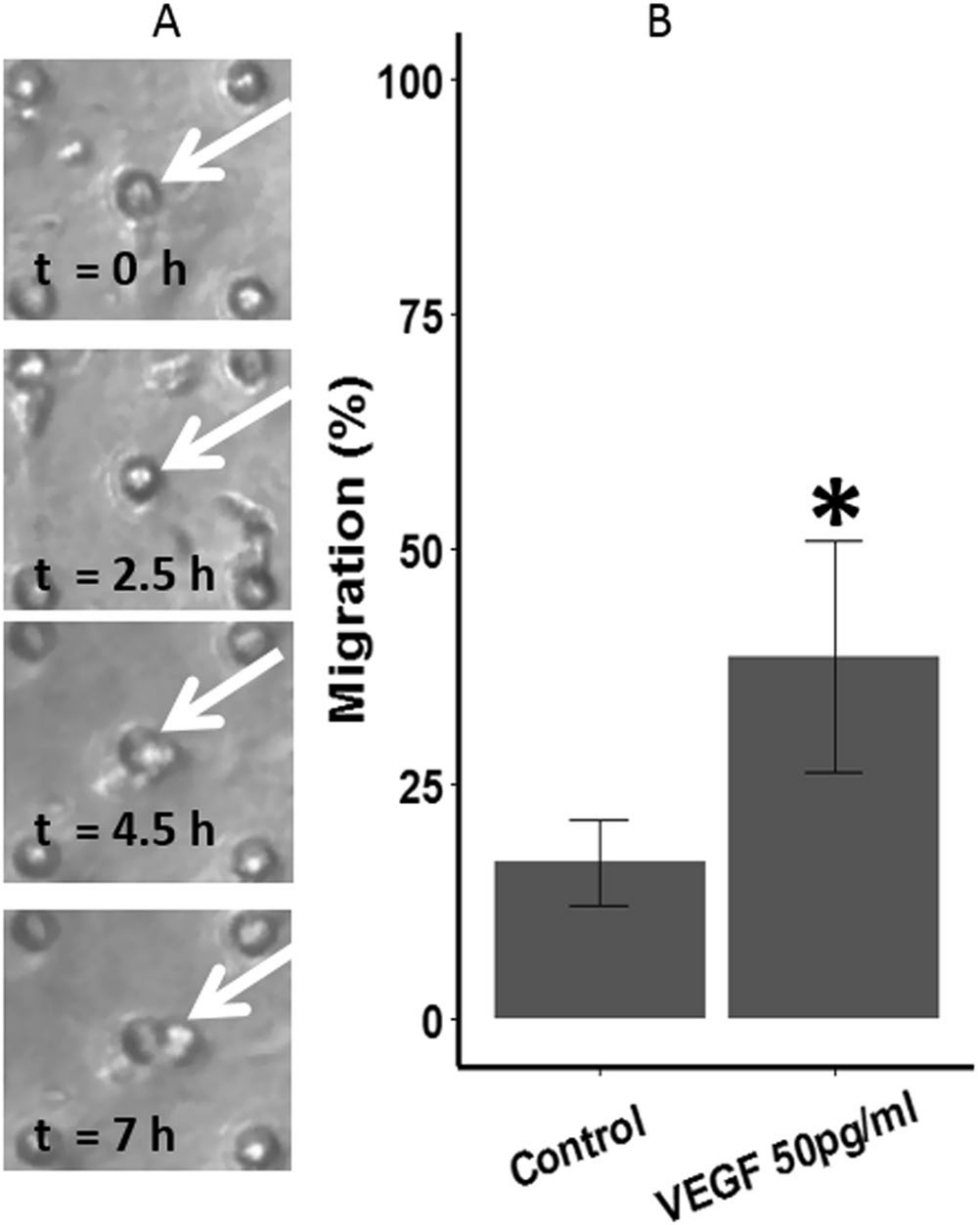
(**A**) Snapshots of HUVEC migrating through the porous membrane taken at different time points. (**B**) Migration (%) of HUVEC at VEGF-free and with VEGF added to the upper channel. Data sets were analysed using Student’s t-test with the asterisk * indicated a significant difference found between the groups (*p = 0.035, n = 6).

The VEGF concentration used in our study (50 pg/ml) was chosen based on the plasma VEGF concentrations from AMD patients quantified in the previous study^32^. Our results indicated that HUVEC responded and moved through the pores toward the source of VEGF (see Movie S1) inside the microfluidic device. Specifically, there was a 21.9% increase in the number of HUVEC migrating through the porous membrane compared to the control where no VEGF was added (Fig. 3).

### Interactions of the co-culture

**Figure 4:**
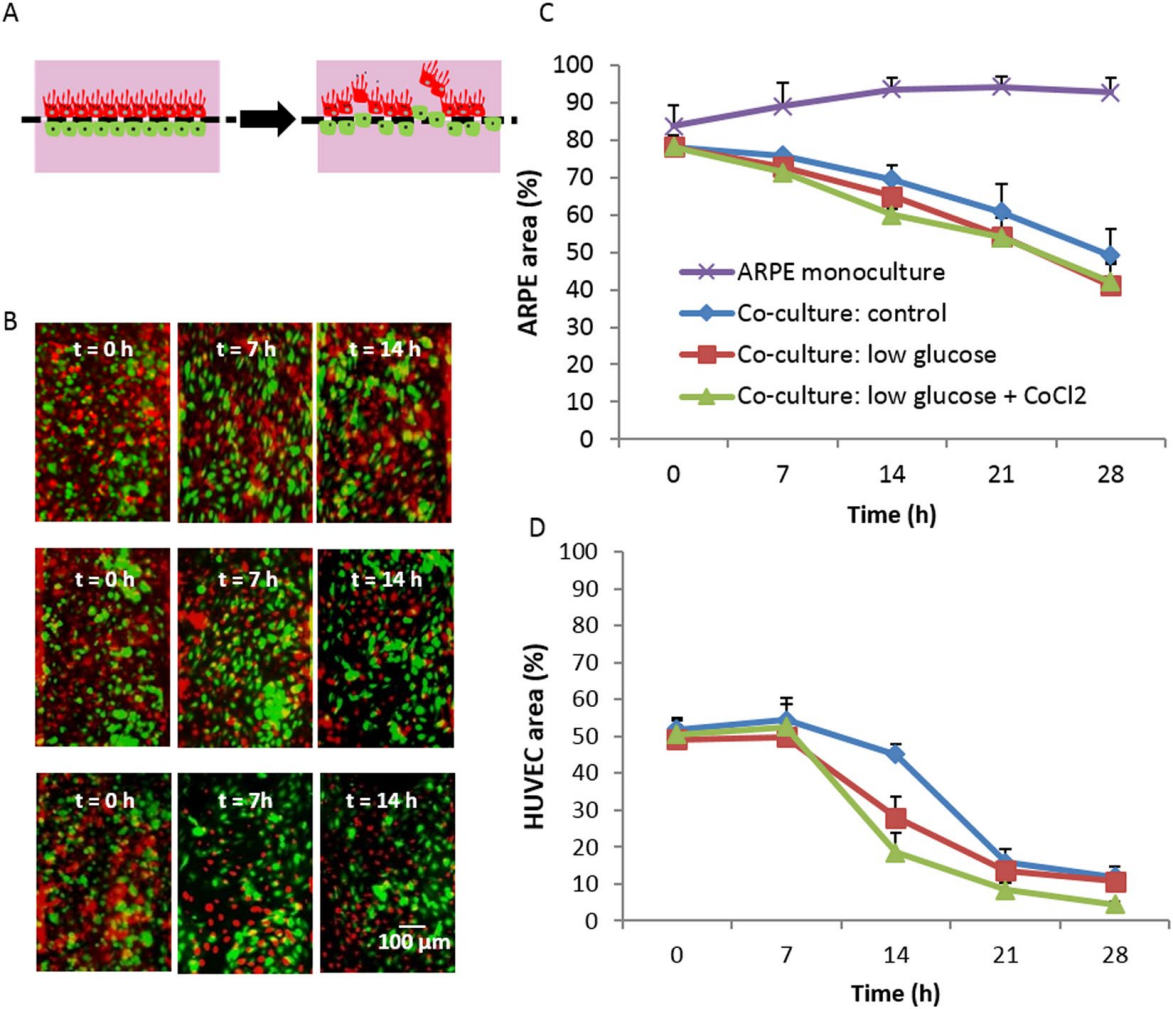
Analysis of the co-culture model. (**A**) Anticipated behaviour of cells within the microfluidic device where ARPE-19 detached due to the invasion of HUVEC; (**B**) Overlaid images of ARPE-19 and HUVEC at different time points; top row: control, middle row: low glucose, bottom row: low glucose with the addition of CoCl_2_; ARPE-19 is depicted in red and HUVEC in green in all cases. (**C**,**D**) Quantification of the ARPE-19 and HUVEC growth area under different medium conditions and monoculture of ARPE in DME High glucose medium. Data were analyzed using 2-way ANOVA tests (showing a significant interaction effect, p < 0.0001 for (**C**) and (**D**)). Significant difference between the ARPE monolayer (purple line) and the co-culture groups (blue, red and green lines) is observed from t = 7 and onwards (p ≤ 0.0001). Pairwise comparisons between co-culture control and low glucose & hypoxia at t = 14 h (p = 0.023), and between all co-culture groups at t = 21 and t = 28 h (p < 0.0001) are significant in (**C**). In (**D**), pairwise comparisons for low glucose and low glucose & hypoxia groups (red and green lines) at t = 14 compared to t = 0 are significant (p ≤ 0.001). N = 7.

Having confirmed the characteristics of individual cells, we investigated if the VEGF produced by ARPE-19 (rather than exogenously supplied) would elicit behavioral changes in HUVEC with co-culture (schematic representation, Fig. 4A). To our best understanding, this phenomenon has neither been proposed nor investigated elsewhere in the literature. As an assessment of the interaction, we overlaid fluorescent images of ARPE-19 and HUVEC at different time points. Judging the results qualitatively (Fig. 4B), HUVEC (green) attached and proliferated the best for a certain time period (approximately 7–10 h after the start of the experiment); the numbers then gradually decreased under all conditions. On the other hand, ARPE-19 cell numbers reduced as the time progressed, with the highest detachment rate 7 hours after the start of the experiments. Figure 4B shows that imposing a combination of low glucose concentration and hypoxia induced the highest loss of ARPE-19 and that the HUVEC grew better (up to certain time point) under such corresponding conditions. We speculate that when ARPE-19 secreted an abnormally higher concentration of VEGF, HUVEC responded by migrating through the pores, causing the detachment of ARPE-19. The whole co-culture experiments was observed for up to 30 h under which HUVEC died as time progressed until the cell density was too low that we terminated the experiments (Fig. 4D). There was also detachment of ARPE-19 post 14 h, but the loss was not as noticeable compared to HUVEC and at least 40% of ARPE-19 survived at the end of experiments (Fig. 4C). In separate experiments (data not shown), we had tested the viability of HUVEC cultured in DMEM (1% FBS) and ARPE-19 cultured in EGM-2 (i.e., swap the culturing media). ARPE-19 proliferated as usual just like those cultured in DMEM; HUVEC could not proliferate under DMEM, however, as they were deprived of many essential supplements required for growth when cultured in DMEM (1% FBS). This leads us into thinking that the media supplied to ARPE-19 diffused into the lower channel and weakened HUVEC, in response to the breakdown of the ARPE-19 monolayer, as the media supplied to ARPE cells had a lower glucose concentration and/or mixed with CoCl_2_.

The observation that HUVEC shape changed from round (t = 0) to a larger, cobblestone-like shape (t = 7) suggested that HUVEC attached and proliferated in the microchannel. From observation, the proliferation rate of HUVEC was slow and gradual (Fig. 4D) unlike other angiogenic studies where high invasion of HUVEC was observed^33^. Based on observations from the permeability testing, ARPE-19 maintained the monolayer under lowered glucose and/or hypoxic conditions. Thus, it suggests that the loss of the ARPE-19 was less likely to be the culturing conditions but due to heterotypic interactions between HUVEC and ARPE-19. In other words, the superimposed images of the co-culture indicate the possible invasion of HUVEC and subsequent detachment of ARPE-19. To quantify these interactions, we further analyzed the area occupied by the ARPE-19 monolayer over time. Figure 4C showed the calculation of the detachment area of ARPE-19 cells (the binary images can be found in Fig. S4). We had characterised the monoculture of HUVEC in order to distinguish the directional migration from the non-directional proliferation of HUVEC. VEGF is one of the first pro-angiogenic factors found, and our results, along with many studies, have proven its effect on HUVEC. Although we did not examine the effect of VEGF antagonists, HUVEC did respond positively to VEGF-A. As the ARPE-19 monolayer in low glucose and/or hypoxic environments secreted 234.84 pg/ml of VEGF from the lower channel which is approximately five times higher than the 50 pg/ml of VEGF that we added to HUVEC monoculture, the directional migration of HUVEC was anticipated.

Combining both results led us to conclude that ARPE-19 secreted excessive VEGF due to the changes in glucose and/or oxygen level which induced directional growth of HUVEC.

There are two prime concerns in the choices of the supplements in the media: (1) whether to mix culture media of each cell line in the co-culture experiments; (2) the use of serum and growth factors in the experimental stage. Different cell types are cultured in their most suitable type of media to optimize the cell growth. Arguably, the medium for the co-culture should be the same to standardise the culturing conditions. However, the composition of a culture medium is optimized to a particular cell type and replacing it for the other could stress cells and deprive them of their essential *elements* therefore exposing them to less than optimal conditions. Previous studies on co-culture and tri-culture had also adopted the same approaches by supplying different cell lines with their respective culture medium^34, 35^, utilizing the advantages offered by the microfluidic systems.

We removed the serum component when conducting ARPE-19 monoculture VEGF analysis as VEGF concentrations of serum could considerably bias the results obtained. For a different reason, we removed the serum in the co-culture experiments; serum starvation has been used as a tool to study various molecular mechanisms such as cellular stress response, protein degradation, apoptosis and to simulate particular pathological conditions^36^ as removal of serum is believed to synchronize the cell cycle^37, 38^. We have adopted this approach to be consistent with previous neovascularization studies^12^.

## Conclusions

We have presented a simple but elegant microfluidic platform where we characterised ARPE-19 and HUVEC monocultures and their co-culture. Utilizing the fact that multi-channels can be segregated from each other within a microfluidic platform, the co-culture platform was used in studying the role of medium conditions on ARPE-19 and the consequent response of HUVEC. Due to limitations mentioned in the discussion above, we did not anticipate observing the complete angiogenesis process but we believe we have taken the first step in elucidating molecular mechanisms of angiogenesis within the microfluidic device. Capabilities of microfluidic systems have been widely applied for years; we seek to further build on the on-chip model of the retina by looking at effects of specific soluble factors and evaluating potential treatments. Because viable RPE cells from AMD patients do not grow well in culture^22^, the process of wound healing appears to be disturbed in AMD patients. In the future, we plan to impose angiogenic regulators such as TNF-alpha on the ARPE-19 monolayer and evaluate the interactions of cells. The consequences of wet AMD are devastating, and much time and effort has been invested in an attempt to better understand the progression of this type of AMD.

Looking at an even broader scope, microfluidics provide customizable platforms to study development of treatments for cancer and other diseases associated with angiogenesis through identification of compounds that stimulate or inhibit the angiogenesis process.

## Methods

### Device fabrication

The designs for the microchannel and micropillars were drawn with SOLIDWORKS (DS SOLIDWORKS) from which silica photomasks with the desired patterns were produced. A master mould was made by spincoating a 100 µm thick layer of SU-8 3050 (Microchem) on a silicon wafer using the photomask to produce the structures by UV-lithography. Similarly, micropillars (Fig. S1) with a height of 30 µm were patterned using the same technique. Microchannels and membranes were made by liquid poly(dimethylsiloxane) (PDMS; Dow Corning Sylgard 184) mixed at a 9:1 ratio of base polymer to curing agent, poured over the SU-8 moulds, degassed under vacuum pressure and cured in a 70 °C oven for at least 3 hours. The cured PDMS were then cut into slabs and removed from the mould. The porous membrane was made from spinning a mixture of PDMS and hexane (2:1, wt%) on micropillars to achieve a thickness of 6.5 µm (pore diameter: 10 µm). A two-layered microfluidic device was made of identical PDMS slabs and a porous membrane, separating the upper and lower microchannel (Fig. 1).

For each device, a PDMS slab was first punched with four 1.5 mm diameter holes for inlets and outlets, bonded irreversibly to the porous membrane via oxygen plasma assisted bonding followed by bonding the second PDMS slab. Upper and lower channels were aligned under a microscope. Once the irreversible bonding was completed, needles were glued firmly to the punched holes of the upper slabs. The device was sterilized under UV light and handled aseptically thereafter.

### Cell cultures

We used ARPE-19 cells kindly provided by Leonard Hjelmeland (Department of Ophthalmology, Section of Molecular and Cellular Biology, University of California, Davis, CA) at passage 10 and cultured in Dulbecco’s Modified Eagle Medium (DMEM) supplemented with 10% fetal bovine serum (FBS) and 1% Antibiotic-Antimycotic (100X, Gibco). Human umbilical vein endothelial cells (HUVEC, Lonza CC-2519) were used in passages 2–8 and cultured in the standard supplemented kit (Lonza, EGM-2 Media). All cultures were maintained at 37 °C in a humidified atmosphere containing 5% CO_2_. Long-term ARPE-19 cultures were cultured in the same conditions mentioned above, but FBS concentration was lowered to 1% until used in experiments.

### Characterisation of cells

Monocultures of ARPE-19 and HUVEC were obtained in order to characterise the cells individually in the microchannel. Before seeding cells into the microfluidic device, the microchannel was coated with poly-L-Lysine solution (0.01%) followed by fibronectin (10 µg/ml). The device was kept in a humidified incubator overnight and filled with either DMEM (10% FBS) or EGM-2 an hour before introducing ARPE-19 and HUVEC, respectively. ARPE-19 were seeded in the upper channel whereas HUVEC were seeded in the lower channel in all cases.

For VEGF analysis, ARPE-19 were cultured alone in the upper channel until they were confluent; medium samples were collected, and VEGF concentration was measured by ELISA (R&D Systems, Minneapolis, MN). The reaction product was quantified with the microplate reader at 450 nm (Fluoroscan Ascent^TM^; Thermo Fisher Scientific, Scotts Valley, CA, USA). To examine the physiologic performance of the epithelial barriers, we adapted the approach executed in a previous study^35^ where the permeability to 70 kDa Fluorescein isothiocyanate-dextran (FITC-dextran, Sigma-Aldrich Chemical Co.) at a concentration of 1 mg∙ml^−1^ was supplied to the upper channel of the ARPE-19 monolayer, and the concentrations were measured (Gemini XPS; Molecular Device) with filters appropriate for 492 nm excitation and 518 nm emission 24 hr after initiation of the perfusion. All results reported here were outputs subtracted from blank values where no dextran was perfused and standardised to the supplied inputs. To support the findings in the FITC-dextran permeability tests, transepithelial electrical resistances (TEER) were also measured to characterise the barrier integrity of ARPE-19 monolayers. Cells were cultured on transwell culture plate inserts (Corning® Transwell®), and TEER was conducted using the commercial system (EVOM 2, World Precision Instruments) and chopstick electrodes (STX-2). All TEER values were determined by subtracting the blank values measured in the absence of cells and standardised to the unit area resistance by multiplying resistance values by the cell culture area. For both VEGF analysis and characterisation of the barrier properties of the APRE, serum-free DMEM High Glucose (20 mM), DMEM Low Glucose (5.5 mM) and DMEM Low Glucose with addition of 150 µM of cobalt(II) chloride (CoCl_2_, Wako) were used to assess monolayers under different conditions where cells in the control group were treated with serum-free DMEM High Glucose while CoCl_2_ was added for the induction of chemical hypoxia. Culturing medium was changed from 1% FBS DMEM to the above conditions for each group 24 hr prior the start of the experiments.

In cell migration analysis, the device was turned upside down to allow HUVEC to adhere to the lower side of the membrane. The device was then turned back to its original orientation to start the monoculture of HUVEC. The upper channel was perfused with 50 pg/ml of VEGF-A_165_ (Wako) and the number of HUVEC migrating to the upper channel was counted manually and was further standardized to the total number of pores present in the area under inspection. Images were recorded using the Real-Time Cultured Cell Monitoring System (CCM-1.4Z, ASTEC) at x100 magnification for 24 hr. The flow rate was set at 50 µl/hr for all the monoculture experiments.

### Staining for fluorescence imaging

As part of the assessment of the growth of cells inside a microfluidic device, ARPE-19 cells were immunostained for the tight junction protein ZO-1. Specifically, ARPE-19 cells in the microchannel were fixed with 3.7% (w/v) formaldehyde (12 min), permeabilized by 0.3% (v/v) Triton X-100 (10 min) and blocked by 5% (w/v) bovine serum albumin (20 min) at room temperature. The sample was incubated with the primary antibody anti-ZO-1 (1:100 dilution; mAb rabbit 40–2300, Invitrogen, CA) overnight at 4 °C. The sample was then stained with Alexa-Fluor 546-conjugated donkey anti-rabbit antibody (1:1000 dilution; A10040, Invitrogen, CA) and incubated at 37 °C (1 hr). Afterwards, the sample was also treated with 4′,6-diamidino-2-phenylindole dihydrochloride (DAPI, Invitrogen, CA) to identify cell nuclei.

For analysis of co-cultures, ARPE-19 and HUVEC were labeled respectively with Cell Tracker^TM^ Fluorescent Probes (Molecular Probes®) red and green (5 µM) according to the manufacturer’s staining protocols.

### Experimental set-up of co-culture

After the staining procedure, cells were allowed to rest and were incubated in their respective media for at least 30 minutes before being loaded into the device. ARPE-19 cells were introduced into the upper channel of the device and cultured until they were confluent. Thereafter, HUVEC were loaded into the lower channel. The device was turned upside down to allow HUVEC to adhere onto the lower side of the membrane. Cells were loaded into the inlet of the channel of interest gently and the other channel was blocked; i.e., ARPE-19 was seeded into the upper microchannel and the inlet and the outlet of the lower channel was blocked during the procedure. HUVEC was seeded in exactly the same manner. This ensures that ARPE and HUVEC were loaded to the microchannel of interest. Cell culture media were supplied to the device by a syringe pump running at 50 µl/hr.

Twenty-four hours prior to the experiment, the respective media were replaced with serum-free versions, and ARPE-19 was exposed to CoCl_2_ (150 µM) in addition to serum-free medium for the hypoxic group. Microscopic images of both HUVEC and ARPE-19 were taken at certain time intervals over 30 h after co-culturing was initiated.

### Image processing

Images were acquired using the fluorescence microscope (Olympus) or the confocal imaging system (LSM700, Carl Zeiss MicroImaging Co., Ltd.) and processed with ImageJ 1.48 v (National Institutes of Health, USA). Corresponding images of ARPE-19 and HUVEC taken at the same time point were first converted to 8-bit before being merged into a colour composite image under the Merge Channels function in ImageJ. To calculate the detachment of ARPE-19 in co-culture experiments, images of the ARPE-19 monolayer at each recorded time point were converted to 8-bit. The area of the channel was selected and cropped. The relative percentage of black and white pixels was then recorded after applying threshold values to the images from which the percentage of detachment was calculated for each sample (Fig. S4).

### Statistical analysis

Quantitative data were expressed as mean ± standard error (SE). When necessary, data were log transformed in order to better meet the assumptions of ANOVA (details of the statistical tests used are indicated in the captions of Fig. 2–4). Tukey’s post-hoc tests were used to evaluate the multiple comparisons among the groups.

All statistical analysis was performed with R version 3.2.5 (R Foundation for Statistical Computing). A level of *p* less than 0.05 was considered to be statistically significant.

## Electronic supplementary material

SUPPLEMENTARY INFO

Movie S1
