# Supplementary material for: Microfluidic co-cultures of retinal pigment epithelial cells and vascular endothelial cells to investigate choroidal angiogenesis
Source: Sci Rep. 2017 Jun 14;7:3538. doi: 10.1038/s41598-017-03788-5 (PMC5471206; doi:10.1038/s41598-017-03788-5)
Supplement: Supplementary file 1 — SUPPLEMENTARY INFO [file 41598_2017_3788_MOESM1_ESM.pdf]

Supporting Information

Microfluidic co-cultures of retinal pigment epithelial cells and vascular endothelial cells to investigate choroidal angiogenesis

Li-Jiun Chen,[a] Shuntaro Ito,[a] Hiroyuki Kai,[a] Kuniaki Nagamine,[a] Nobuhiro Nagai,[b] Matsuhiko Nishizawa,[a] Toshiaki Abe,[b] and Hirokazu Kaji*[a]

[a] *Department of Finemechanics, Graduate School of Engineering, Tohoku University, Japan. E-mail: kaji@biomems.mech.tohoku.ac.jp*

[b] *Division of Clinical Cell Therapy, United Centers for Advanced Research and Translational Medicine, Tohoku University Graduate School of Medicine, Japan*

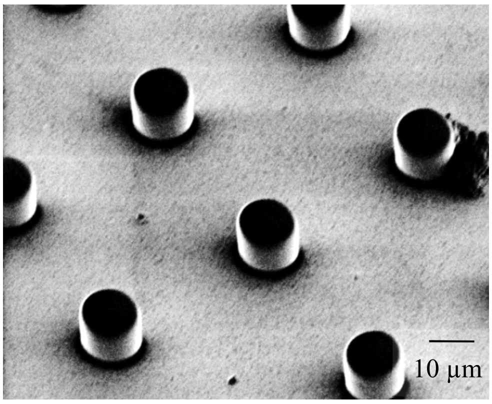

Fig. S1. SEM image (inclined 30 degrees) of micropillars on which PDMS was spin-coated to make porous membranes. Height of pillars is about 30 µm.

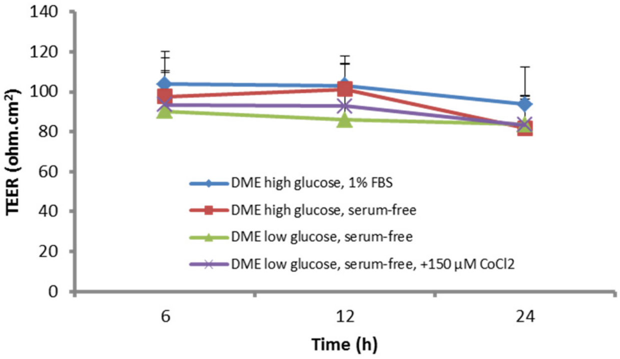

Fig. S2. Effect of time on TEER of ARPE-19 monoculture in the transwell culture plate inserts. No significant difference between different treatments were found at time = 24 h. Time axis showed the time passed since the initiation of the experiment. Standard error bars represent one standard deviation from the mean.

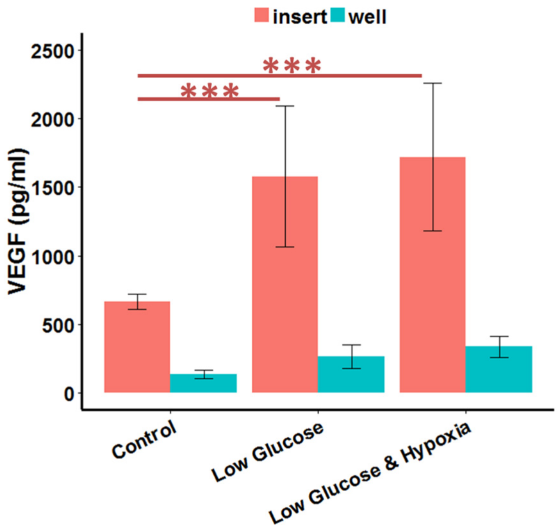

Fig. S3. VEGF-analysis of monoculture of ARPE-19 cultured in the transwell culture plate inserts. This data is meant to supplement the VEGF analysis in microfluidic devices where insert corresponds to the upper channel and well corresponds to the lower channel. Experimental conditions were the same as experiments performed in microfluidic devices (see also Fig. 2E). Asterisks * are indicated for pairwise comparisons of upper channels between experimental conditions (***$p \leq 0.0001$)); pairwise comparisons among an experimental condition (red and blue bars) for all experimental conditions are significant ($p < 0.0001$). N = 8.

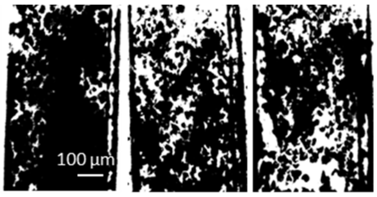

Time (h)	t = 0	t = 7	t = 14
Black:white pixel (%)	80.50	72.18	68.73

Fig. S4. Representative binary images of ARPE monolayer in serum-free DMEM High Glucose medium. Black area indicates the presence of cells while white space represents area where no cells are present. Percentages of black to white pixels were calculated and used to make the graph in Fig. 4C.

Movie S1. HUVEC migrating through the porous membrane.

HUVEC was cultured on the lower side of the membrane inside a microfluidic device with 50 pg/ml of VEGF-A_{165} supplied in the upper channel. Directional migration occurred when cells migrated across the pores (details were described in ‘Characterization of cells’ under Methods section). Images were recorded and used to make the video at 6 FPS in ImageJ.
